# Low-Density Lipoprotein Cholesterol and Mortality in Patients With Intracerebral Hemorrhage in Taiwan

**DOI:** 10.3389/fneur.2021.793471

**Published:** 2022-01-17

**Authors:** Chi-Pang Wen, Yi-Che Lee, Yuan-Ting Sun, Chih-Yuan Huang, Chon-Haw Tsai, Po-Lin Chen, Wei-Lun Chang, Po-Yen Yeh, Cheng-Yu Wei, Ming-Jun Tsai, Yu Sun, Chih-Hao Lin, Jiunn-Tay Lee, Ta-Chang Lai, Li-Ming Lien, Mei-Chen Lin, Cheng-Li Lin, June-Han Lee, Hao-Kuang Wang, Chung Y. Hsu, Taiwan Stroke Registry Investigators, Chung-Hsiang Liu

**Affiliations:** ^1^Graduate Institute of Biomedical Sciences, College of Medicine, China Medical University, Taichung, Taiwan; ^2^Department of Medical Research, China Medical University Hospital, Taichung, Taiwan; ^3^Institute of Population Health Sciences, National Health Research Institutes, Zhunan, Taiwan; ^4^School of Medicine, I-Shou University, Kaohsiung, Taiwan; ^5^Department of Nephrology, E-Da Hospital, Kaohsiung, Taiwan; ^6^Department of Neurology, College of Medicine, National Cheng Kung University Hospital, National Cheng Kung University, Tainan, Taiwan; ^7^Department of Surgery, Faculty of Neurosurgical Service, National Cheng Kung University Hospital, Tainan, Taiwan; ^8^Division of Neurology, China Medical University Hospital, Taichung, Taiwan; ^9^Neurological Institute, Taichung Veterans General Hospital, Taichung, Taiwan; ^10^Department of Neurology, Show Chwan Memorial Hospital, Changhua, Taiwan; ^11^Department of Neurology, St. Martin De Porres Hospital, Chiayi, Taiwan; ^12^Department of Neurology, Chang Bing Show Chwan Memorial Hospital, Changhua, Taiwan; ^13^Department of Neurology, Tainan Municipal An-Nan Hospital-China Medical University, Tainan, Taiwan; ^14^Department of Neurology, En Chu Kong Hospital, New Taipei City, Taiwan; ^15^Department of Neurology, Lin Shin Hospital, Taichung, Taiwan; ^16^Department of Neurology, Tri-Service General Hospital, National Defense Medical Center, New Taipei City, Taiwan; ^17^Department of Neurology, Cheng Hsin General Hospital, New Taipei City, Taiwan; ^18^Department of Neurology, Shin Kong Wu Ho Su Memorial Hospital, New Taipei City, Taiwan; ^19^Management Office for Health Data, China Medical University Hospital, Taichung, Taiwan; ^20^College of Medicine, China Medical University, Taichung, Taiwan; ^21^Department of Neurosurgery, E-Da Hospital, Kaohsiung, Taiwan; ^22^Department of Neurology, China Medical University Hospital, Taichung, Taiwan; ^23^Graduate Institute of Clinical Medical Science, China Medical University, Taichung, Taiwan; ^24^Taiwan Stroke Society, New Taipei City, Taiwan

**Keywords:** stroke, ICH, LDL, Taiwan Stroke Registry, mortality, proportional hazards regression analysis

## Abstract

**Objective:** Lower serum low-density lipoprotein cholesterol (LDL-C) levels are associated with increased intracerebral hemorrhage (ICH) risk. However, reverse causality and residual confounding has not attracted public attention. Therefore, we assessed whether people with LDL-C have increased risk of mortality adjusting for potential confounders using two large Taiwan cohorts.

**Methods:** The Mei-Jhao (MJ) cohort has 414,372 adults participating in a medical screening program with 378 ICH deaths within 15 years of follow-up (1994–2008). Cox proportional hazards regressions estimated hazard death ratios according to LDL-C levels. We identified 4,606 ICH patients from the Taiwan Stroke Registry (TSR) and analyzed the impact of LDL-C on 3-month mortality.

**Results:** Low cholesterol (LDL-C <100 mg/dL), found in 1/4 of the MJ cohort, was highly prevalent (36%) among young adults (age 20–39). There was a graded relationship between cholesterol and mortality for ICH [Hazard ratio, 1.56; 95% confidence interval (CI), 1.13–2.16]. Compared with patients with an LDL-C of 110–129 mg/dL in TSR, the risk for mortality was 1.84 (95% CI, 1.28–2.63) with an LDL-C of <100 mg/dL.

**Conclusion:** Lower serum LDL-C level independently predicts higher mortality after acute ICH. While its causative role may vary, low cholesterol may pose potential harms in Taiwan.

## Introduction

Stroke is the second commonest cause of death worldwide after ischemic heart disease, accounting for 6 million deaths in 2016 (1). Strokes can be broadly classified as hemorrhagic or ischemic. Intracerebral hemorrhage (ICH) accounts for only 10–15% of all strokes. ICH occurs following the rupture of cerebral vessels, usually manifesting as a rapidly expanding hematoma arising within the brain parenchyma. It is significantly associated with a worse functional outcome and higher mortality compared with ischemic stroke (2). Given the increasing burden and costs associated with stroke care, there is a pressing need to identify potential modifiable risk factors (3, 4).

Low-density lipoprotein cholesterol (LDL-C) transfers lipids around the body in the extracellular fluid, making them available to the body's cells for receptor-mediated endocytosis. LDL-C can contribute to atherosclerosis if it is oxidized within the walls of arteries. Therefore, LDL-C has long been associated with the risk for ischemic stroke, and myocardial infarction (5). A reduction in LDL-C level has been demonstrated to reduce cardiovascular disease risk significantly.

Some studies have shown a relationship between low cholesterol levels and hemorrhagic stroke (6). However, those observational studies were pretreated with statins (7, 8). Neurologists are aware that statins may increase the risk for future ICH but have focused mainly on statins safety (7, 8). However, the link between LDL-C levels, ICH mortality, and clinical deterioration in patients with acute ICH remains largely unknown.

We assessed the mortality rates of low low-density lipoprotein cholesterol (LDL-C) and cross-tabulated their overlapping effects in a large cohort sample. To gain a clearer picture of ICH developments, we have included an additional prospective Taiwan Stroke Registry (TSR) cohort. This cohort has accumulated >100,000 stroke cases (and counting) and has records of hospital stay and follow-up information on mortality. With these two large data sets, we examined the impact of lower LDL on ICH mortality by analyzing stroke and healthy adult data together.

## Methods

### Mei-Jhao Health Survey Data

#### Study Population and Data Collection

The MJ cohort consisted of adults aged 20 years or older who participated in a self-paying health surveillance program between 1994 and 2008. A description of this cohort has been reported previously (9, 10). The data used in this research were authorized by and received from MJ Health Research Foundation (Authorization Code: MJHRFB2014001C). Any interpretation or conclusion described in this paper does not represent the views of MJ Health Research Foundation. All participants in the research have given written informed consent before the health examination to authorize the data analysis. Personal identification data was removed in the MJ Health Research Foundation, so the participants remained anonymous during the whole research process. The detail of the study population and data collection is described and reported elsewhere (http://www.mjhrf.org/main/page/resource/en/#resource04). The study protocol conformed to the ethical standards established by the Declaration of Helsinki (1964), which do not require written or verbal consent for data-linkage studies.

#### Main Outcome Measures

This study consisted of 414,372 adults with a median follow-up of 8.62 years. Those with cancer history were excluded. Each participant went through a standard panel of medical screening with fasting blood analyzed by a Hitachi 7150 Autoanalyzer, and a self-administered history and lifestyle questionnaire. LDL-C was measured directly in the laboratory. Vital status was identified by matching with the Taiwan death file: a total of 11,787 deaths were identified. Taiwan death file were categorized as per the diagnosis codes assigned by the International Classification of Diseases, 10th Revision Clinical Modification (ICD-10-CM); All-cause mortality (A00-Y98), Coronary heart disease (I01-I02.0, I05-I09, I20-I25, I27, I30-I52), Intracerebral hemorrhage (I60-I62), and Ischemic stroke (I63-I69) were used in the study.

#### Statistical Analysis

Cox proportional hazards models were used to assess LDL-C and TC associations with all-cause and cause-specific mortality. Hazard ratios (HRs) and corresponding 95% confidence intervals (CIs) were estimated for mortality risk using LDL-C and TC as categorical variables. LDL-C and TC also were regarded as continuous variables to estimate the effect per 1 mg/dL decrease in level among those with an LDL-C level lower than 110 mg/dL or TC level lower than 180 mg/dL. Low cholesterol was defined as LDL-C <100 mg/dL or TC <160 mg/dL, and very low cholesterol as LDL-C <70 mg/dL or TC <130 mg/dL. HRs were adjusted for 9 variables: age, gender, body mass index (BMI), systolic blood pressure (SBP), fasting glucose level, smoking status, alcohol consumption, physical activity, and anemia status. Sensitivity analyses were conducted by excluding participants who: died within the first 5 or 9 years of follow-up; were enrolled for <1 year, were carriers of hepatitis B virus (HBV) or hepatitis C virus (HCV), had liver diseases (liver cancer and liver cirrhosis), had abnormal liver functions [alanine aminotransferase (ALT) >40 U/L or aspartate aminotransferase (AST) >40 U/L], or had a history of cardiovascular disease (CVD), diabetes, liver cirrhosis, or kidney disease at enrollment. In addition, stratifying analysis of all-cause mortality risk by demographic, behavioral, and medical characteristics was performed. We used Markov chain Monte Carlo multiple imputation with 10 iterations to account for missing observations. We assumed that the imputation model had a joint multivariate normal distribution, and imputed data calculations were only performed on sensitivity analysis in hepatitis virus infection. Life expectancy was calculated using the Chiang's life table method. All analyses were performed with Stata (College Station, TX), and 2-sided *p* < 0.05 was considered statistically significant.

### Taiwan Stroke Registry

#### Data, Analytic Methods, and Research Materials Transparency

The Taiwan Stroke Registry (TSR) is a nationwide hospital-based prospective study with 56 participating stroke centers. The study was established in 2006 and is sponsored by the Taiwan Department of Health (11). Ethical approval for the study was obtained from China Medical University and the Institutional Review Boards (IRB) of the collaborating hospitals (CMUH104-REC2-115). Details on the database's generation, monitoring, and maintenance are published by the Taiwan Stroke Society (12, 13). All participants provided informed consent. The TSR provides a structured record of demographics, including stroke types, imaging, in-hospital management, other known vascular risk factors, and long-term functional outcomes at follow-up between August 1st, 2006 and May 20th, 2016. Patients were included in the present study if they met the following criteria: (1) had suffered from a stroke and presented at the hospital within 10 days of symptom onset, and (2) were diagnosed with stroke as confirmed by a neurologist or neurosurgeon.

#### Main Outcome Measures

ICH patients with missing LDL-C data were excluded from the study. Patients were categorized into six groups based on their baseline levels: <70, 70–99, 100–129, 130–159, and ≥160 mg/dL of LDL-C. The primary factors considered in the present study were the potential confounding factors. For each case, stroke severity was determined using the National Institutes of Health Stroke Scale (NIHSS) scores. Modified Rankin Scales (mRS) were used to assess functional outcomes. A mRS score of 3 or higher was considered an unfavorable outcome. Vascular risk factors were defined in accordance with the consensus TSR criteria. Serum LDL-C were obtained within the first 24 h from symptom onset after a minimum of 12 h fasting. All registered follow-up data were collected from patients at 3-month after stroke. This data was collected either in the clinic or *via* telephone interview for those who could not be present at the clinic. Follow-up data included assessment of functional status, stroke recurrence, and survival.

#### Statistical Analysis

Data analysis first compared distributions of age, sex, stroke risk factors, medications, and laboratory data. A Cox proportional hazard regression was used to estimate the risk of mortality. Univariate and multivariate logistic regression analyses were used to determine crude odds ratios (OR) and adjusted odds ratios (aOR), by several variables: Age, gender, past history, medication, NIHSS score, hematoma condition, and surgery. Patients with LDL-C 100–129 mg/dL was used as the reference group. The threshold for statistical significance was set at a two-sided *p*-value of ≤0.05.

## Results

### Low LDL Increase ICH Mortality in MJ Cohort

**Figure 1:**
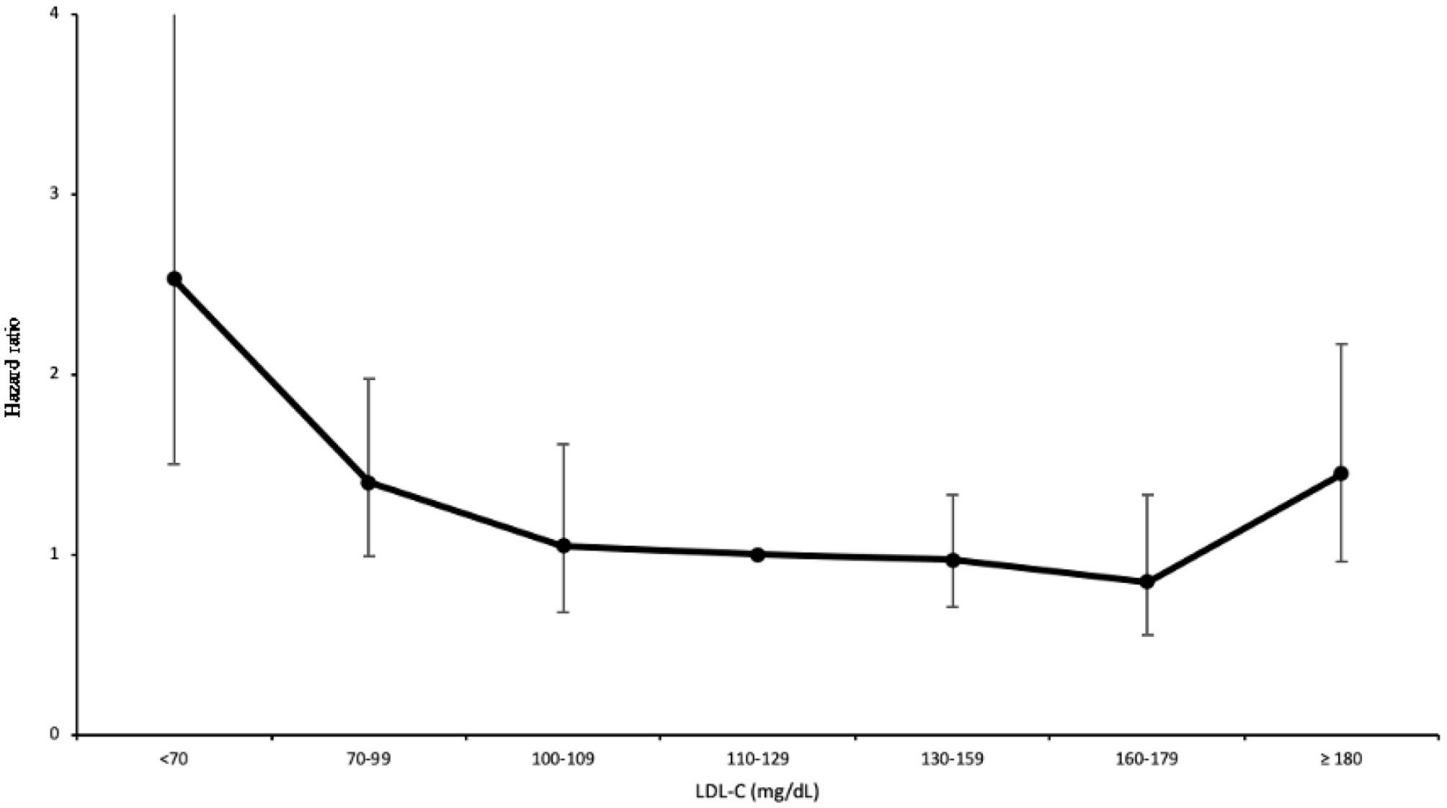
LDL-C and hazard ratios for mortality from ICH in MJ cohort.

The MJ cohort consisted of 414,372 adults with a median follow-up of 8.62 years. The vital status was identified by matching with the Taiwan death file. A total of 11,787 deaths were identified (378 deaths were ICH related). One-quarter of the cohort (28%) and one-third of younger people (age 20–39) (37%) had low LDL-C (<100 mg/dL). Nearly half of the underweight patients (BMI <18.5 kg/m^2^) had low cholesterol (49%) (Table 1). After controlling for 9 confounding factors, a U-shaped association was found between LDL levels and all-cause mortality (Figure 1). Compared to their counterpart in the range of 110–129 mg/dL (LDL-C), participants with low LDL-C (<100 mg/dL) had a 30% increase in all-cause mortality (Table 2). Furthermore, among low cholesterol participants we found a significant increase of mortality (~56%) for ICH, contributing to increases in cardiovascular disease (CVD) mortality. Coronary heart disease did not increase at low cholesterol, with a J-shaped association, confirming an HR at 2.54 for high LDL-C ≥180 mg/dL.

**Table 1:** Distribution of characteristics by LDL-C levels in MJ cohort.

		**LDL-C levels (mg/dL), %^†^**								
All, %^*^ N **=** **414,372**			**<100**	**<70**	**70–99**	**100–109**	**110–129**	**130–159**	**160–179**	**≥180**
			28.1	4.3	23.8	12.4	24.5	23.6	6.7	4.6
Age, years	20–39	54.5	35.7	5.7	30.0	14.0	24.5	19.1	4.2	2.5
	40–64	38.5	19.5	2.7	16.8	10.7	24.6	28.9	9.4	6.9
	≥65	7.0	17.1	2.3	14.7	9.6	23.9	30.1	10.7	8.6
Gender	Men	47.8	23.6	3.4	20.2	11.7	25.3	26.6	7.7	5.1
	Women	52.2	32.3	5.2	27.1	13.1	23.7	20.9	5.7	4.2
Smoking status	Non-smoker	70.7	29.8	4.6	25.2	12.8	24.4	22.6	6.2	4.3
	Ex-smoker	6.1	22.2	3.0	19.2	11.0	24.7	27.5	8.5	6.1
	Current-smoker	23.2	25.8	4.2	21.6	12.0	24.6	25.3	7.4	5.0
Drinking	Non-drinker	78.2	29.3	4.4	24.9	12.7	24.4	22.8	6.3	4.4
	Occasional drinker	3.0	25.3	3.7	21.5	11.3	23.9	25.4	7.9	6.1
	Regular drinker	18.8	25.0	4.2	20.8	11.6	24.7	26.0	7.6	5.1
Physical activity	Inactive	52.9	29.6	4.7	24.9	12.6	24.3	22.7	6.3	4.5
	Low active	22.0	28.5	4.3	24.2	12.7	24.9	23.2	6.5	4.3
	Fully active	25.1	24.7	3.6	21.1	11.8	24.6	25.9	7.7	5.3
BMI (kg/m^2^)	<18.5	8.3	49.2	9.9	39.3	14.8	20.0	12.2	2.4	1.4
	18.5–24.9	65.0	29.4	4.4	25.0	13.0	24.8	22.7	6.0	4.1
	25–29.9	22.9	18.5	2.4	16.1	10.4	25.0	29.5	9.6	7.0
	≥30	3.8	18.6	2.8	15.8	10.6	24.9	29.8	9.3	6.9
SBP (mmHg)	<120	50.7	34.0	5.5	28.6	13.7	24.4	20.0	4.8	3.0
	120–139	29.9	24.5	3.5	21.1	12.0	25.1	25.8	7.4	5.1
	≥140 or HTN	19.5	18.1	2.7	15.5	9.9	23.7	29.8	10.3	8.1
Fasting glucose (mg/dL)	<110	89.3	29.2	4.5	24.8	12.8	24.6	23.0	6.2	4.2
	110–125	5.6	17.7	2.9	14.8	9.6	23.4	30.3	10.7	8.3
	≥126 or DM	5.1	20.2	3.5	16.7	9.5	22.7	28.1	10.4	9.1
Anemia^‡^	No	92.5	27.1	4.0	23.1	12.3	24.7	24.2	6.9	4.8
	Yes	7.5	41.4	8.1	33.3	14.0	21.9	16.2	3.9	2.5
Hepatitis virus infection^§^	HBV(–) & HCV(–)	62.6	27.3	4.0	23.2	12.5	24.7	24.2	6.8	4.6
	HBV(+) or HCV(+)	37.4	27.4	3.9	23.6	13.0	25.5	23.8	6.3	3.9

*LDL-C, low-density lipoprotein cholesterol; TC, total cholesterol; BMI, body mass index; SBP, systolic blood pressure; HTN, hypertension; DM, diabetes; HBV, hepatitis B virus; HCV, hepatitis C virus. To convert mg/dL to mmol/L, multiply by 0.0259*.

* *Percentages by column*.

† *Percentages by row*.

‡ *Anemia is defined as hemoglobin level <13 g/dL for men and <12 g/dL for women*.

§ *Missing for 257,498 (62.1%) participants*.

**Table 2:** Multivariable adjusted hazard ratios for all-cause and cause-specific mortality by LDL-C levels in MJ cohort.

	**No. of cases**		**LDL-C levels (mg/dL)**														
		** <100**		** <70**		**70–99**		**100–109**		**110–129**		**130–159**		**160–179**		**≥180**	
		***n***	**HR (95% CI)**	***n***	**HR (95% CI)**	***n***	**HR (95% CI)**	***n***	**HR (95% CI)**	***n***	**HR**	***n***	**HR (95% CI)**	***n***	**HR (95% CI)**	***n***	**HR (95% CI)**
All-cause mortality	11,787	2,521	1.30^*^ (1.23–1.38)	461	1.72^*^ (1.54–1.92)	2,060	1.24^*^ (1.16–1.32)	1,192	1.11^*^ (1.03–1.20)	2,639	ref	3,193	0.97 (0.91–1.02)	1,163	0.96 (0.89–1.04)	1,079	1.13^*^ (1.04–1.22)
CVD	2,236	357	1.16^*^ (1.00–1.35)	66	1.64^*^ (1.24–2.17)	291	1.09 (0.92–1.28)	200	1.11 (0.92–1.33)	447	ref	686	1.18^*^ (1.03–1.34)	241	1.16 (0.98–1.38)	305	1.80^*^ (1.53–2.11)
CHD	614	71	0.81 (0.59–1.12)	14	1.14 (0.61–2.12)	57	0.76 (0.54–1.08)	49	1.01 (0.71–1.45)	120	ref	187	1.24 (0.97–1.59)	78	1.48^*^ (1.09–2.01)	109	2.54^*^ (1.92–3.37)
Ischemic stroke	553	79	0.94 (0.69–1.28)	10	1.03 (0.52–2.04)	69	0.92 (0.67–1.28)	50	0.99 (0.69–1.41)	123	ref	181	1.03 (0.80–1.33)	54	0.91 (0.64–1.28)	66	1.32 (0.95–1.84)
ICH	378	86	1.56^*^ (1.13–2.16)	18	2.53^*^ (1.50–4.26)	68	1.40 (0.99–1.98)	37	1.05 (0.68–1.61)	82	ref	102	0.97 (0.71–1.33)	32	0.85 (0.55–1.33)	39	1.45 (0.96–2.17)

*LDL-C, low-density lipoprotein cholesterol; CVD, cardiovascular disease; CHD, coronary heart disease; ICH, intracerebral hemorrhage; HR, hazard ratio; CI, confidence interval. To convert mg/dL to mmol/L, multiply by 0.0259*.

* *Indicates a significant (p < 0.05) death rate compared to LDL-C levels between 110 and 129 mg/dL. Hazard ratio adjusted for age, gender, body mass index, smoking status, alcohol consumption, physical activity, systolic blood pressure, fasting glucose level, and anemia*.

### Low LDL Had Higher Mortality in TSR

**Figure 2:**
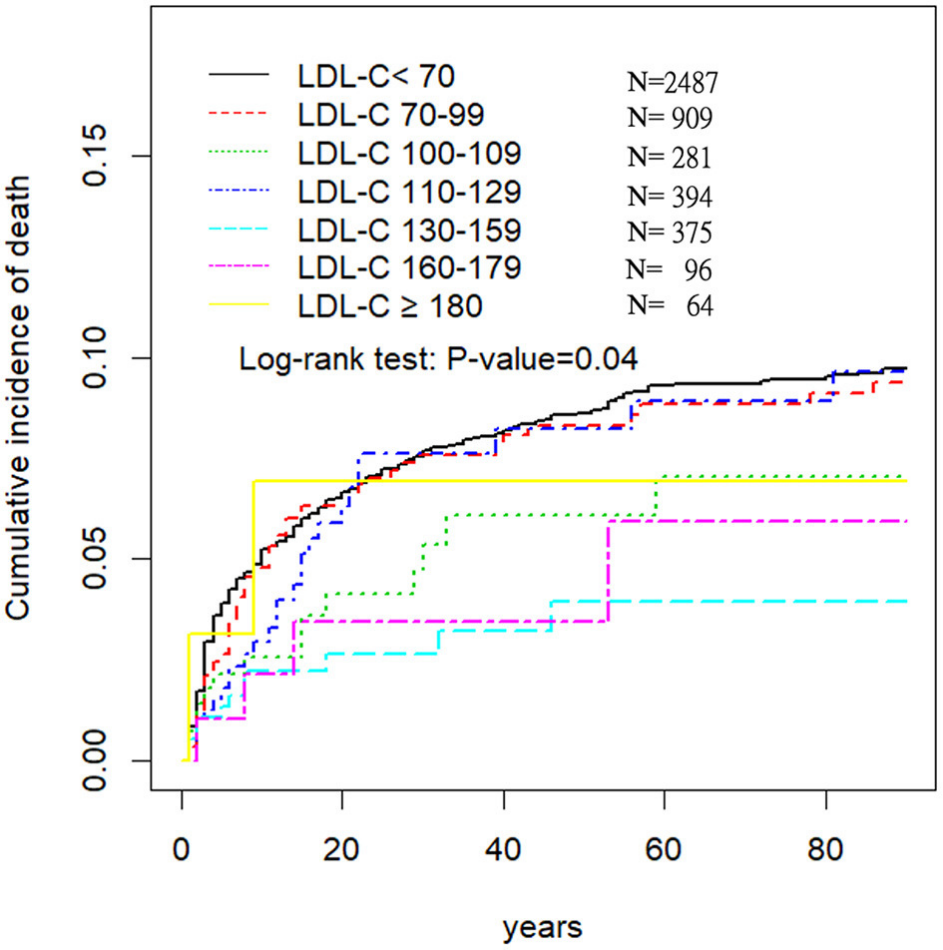
Three-month survival in different LDL-C groups in 4,066 ICH patients.

We excluded 9,037 patients without fasting TC and 581 patients without 3 months follow up records from the TSR. Hence, in this study we included 4,606 spontaneous ICH patients with baseline fasting LDL levels measured immediately after stroke. The mean (SD) age was 61.7 (51.9–72.8) years, and 1,519 (33.0%) were female (Table 3, Supplementary Table 1). Patients with LDL <70 mg/dL were older and mainly male (Table 3, Supplementary Table 2). However, there were no obvious differences in pre-ICH medications (Supplementary Table 3). Patients on admission presented with a median GCS score of 15 [interquartile range (IQR), 11 ± 15] and a median NIHSS score of 9 (IQR, 3 ± 18). The overall case-mortality rate of ICH at 3-month was 7.56%. At 3-month, most patients (70%) had a mRS of >2. Patients with LDL <70 mg/dL were more likely to have hematoma enlargement, and a higher 3-month mortality (Table 4). Finally, compared to patients with LDL 110–129 mg/dL, those with LDL <100 mg/dL had higher 3-month mortality (aOR, 1.84; 95% CI, 1.28–2.63) and initial NIHSS >15 (aOR, 1.35 95% CI, 1.13–1.62) (Table 5, Figure 2).

**Table 3:** Baseline characteristics of the study population in TSR, *n* (%).

	**Total**	**LDL-C**								
	***N* = 4,606**	** <100** ***N* = 3,396**	** <70** ***N* = 2,487**	**70–99** ***N* = 909**	**100–109** ***N* = 281**	**110–129** ***N* = 394**	**130–159** ***N* = 375**	**160–179** ***N* = 96**	**≥180** ***N* = 64**	***P*-value**
Sex										0.39
Women	1,519 (33.0)	1,088 (32.0)	803 (32.3)	285 (31.4)	99 (35.2)	137 (34.8)	139 (37.1)	32 (33.3)	24 (37.5)	
Men	3,087 (67.0)	2,308 (68.0)	1,684 (67.7)	624 (68.7)	182 (64.8)	257 (65.2)	236 (62.9)	64 (66.7)	40 (62.5)	
Age (years)	61.7	62.4	62.3	62.6	59.8	61.4	59.6	55.6	53.7	<0.001
Hypertension	3,823 (83.0)	2,728 (80.3)	1,963 (78.9)	765 (84.2)	246 (87.5)	351 (89.1)	350 (93.3)	87 (90.6)	61 (95.3)	<0.001
Diabetes	1,129 (24.5)	858 (25.3)	608 (24.5)	250 (27.5)	56 (19.9)	90 (22.8)	92 (24.5)	16 (16.7)	17 (26.6)	0.07
Heart disease	693 (15.1)	543 (16.0)	410 (16.5)	133 (14.6)	36 (12.8)	57 (14.5)	44 (11.7)	7 (7.29)	6 (9.38)	0.02
Previous stroke	793 (17.2)	607 (17.9)	448 (18.0)	159 (17.5)	39 (13.9)	71 (18.0)	53 (14.1)	14 (14.6)	9 (14.1)	0.33
Uremia	113 (2.95)	92 (3.23)	69 (3.22)	23 (3.25)	5 (2.20)	10 (3.22)	4 (1.27)	1 (1.35)	1 (1.85)	0.50
Alcoholism	1,003 (21.8)	749 (22.1)	499 (20.1)	250 (27.5)	69 (24.6)	87 (22.1)	64 (17.1)	17 (17.7)	17 (26.6)	<0.001
Smoking	1,617 (37.8)	1,174 (37.8)	801 (36.6)	373 (43.4)	101 (37.4)	162 (43.1)	111 (30.5)	35 (38.0)	34 (53.1)	<0.001

**Table 4:** Condition of the study population in TSR, *n* (%).

	**Total**	**LDL-C**								
		** <100**	** <70**	**70–99**	**100–109**	**110–129**	**130–159**	**160–179**	**≥180**	***P*-value**
NIHSS score upon admission										<0.001
1–5	1,341 (29.1)	933 (27.5)	685 (27.5)	248 (27.3)	84 (29.9)	137 (34.8)	136 (36.3)	33 (34.4)	18 (28.1)	
6–10	895 (19.4)	642 (18.9)	475 (19.1)	167 (18.4)	68 (24.2)	81 (20.6)	73 (19.5)	20 (20.8)	11 (17.2)	
11–15	689 (15.0)	502 (14.8)	363 (14.6)	139 (15.3)	41 (14.6)	59 (15.0)	57 (15.2)	16 (16.7)	14 (21.9)	
16–20	388 (8.42)	308 (9.07)	229 (9.21)	79 (8.69)	25 (8.90)	16 (4.06)	28 (7.47)	7 (7.29)	4 (6.25)	
>20	929 (20.2)	715 (21.1)	493 (19.8)	222 (24.4)	47 (16.7)	75 (19.0)	62 (16.5)	19 (19.8)	11 (17.2)	
Median	9 (3–18)	9 (3.5–18)	9 (3–18)	10 (4–20)	8 (4–16)	7 (3–14)	7 (3–15)	9 (4.5–16)	10 (4–15)	0.004
Initial GCS										0.01
3–5	190 (4.16)	143 (4.24)	96 (3.89)	47 (5.21)	15 (5.40)	14 (3.57)	11 (2.96)	3 (3.16)	4 (6.35)	
6–8	428 (9.37)	350 (10.4)	247 (1.0)	103 (11.4)	18 (6.47)	27 (6.89)	24 (6.45)	5 (5.26)	4 (6.35)	
9–12	783 (17.1)	595 (17.7)	446 (18.1)	149 (16.5)	35 (12.6)	66 (16.8)	58 (15.6)	19 (20.0)	10 (15.9)	
13–15	3,168 (69.3)	2,281 (67.7)	1,678 (68.0)	603 (66.9)	210 (75.5)	285 (72.7)	279 (75.0)	68 (71.6)	45 (71.4)	
Median	15 (11–15)	15 (11–15)	15 (11–15)	15 (11–15)	15 (13.−15)	15 (12–15)	15 (13–15)	15 (11–15)	15 (12–15)	0.005
Hematoma enlargement	170 (3.69)	139 (4.09)	117 (4.70)	22 (2.42)	8 (2.85)	12 (3.05)	6 (1.60)	3 (3.13)	2 (3.13)	0.009
Surgery for ICH	681 (14.8)	509 (15.0)	350 (14.1)	159 (17.5)	48 (17.1)	58 (14.7)	47 (12.5)	11 (11.5)	8 (12.5)	0.12
Outcome at 3 months										
Death	348 (7.56)	289 (8.51)	222 (8.93)	67 (7.37)	14 (4.98)	26 (6.60)	11 (2.93)	4 (4.17)	4 (6.25)	0.001
mRS >2	3,222 (70.0)	2,383 (70.2)	1,712 (68.8)	671 (73.8)	206 (73.3)	270 (68.5)	251 (66.9)	68 (70.8)	44 (68.8)	0.07

**Table 5:** Association between initial stroke severity and 3 months outcome by LDL-C levels and pre-ICH lipid-lowering drugs use in TSR.

	**OR (95% CI)**		
**All ICH patients** **(*n* = 4,606)**	**Crude**	**Age/sex-adjusted**	**Multivariate adjusted**
**3-month mortality**			
<100	1.39 (0.91, 2.11)	1.35 (0.88, 2.05)	1.84 (1.28, 2.63)
<70	1.82 (1.36, 2.42)	1.75 (1.31, 2.34)	1.33 (0.79, 2.23)
70–99	1.13 (0.71, 1.80)	1.10 (0.69, 1.76)	1.21 (0.68, 2.14)
100–109	0.74 (0.38, 1.45)	0.74 (0.38, 1.44)	0.71 (0.31,1.63)
110–129	1	1	1
130–159	0.43 (0.21, 0.88)	0.42 (0.20, 0.87)	0.38 (0.15,0.95)
160–179	0.62 (0.21, 1.81)	0.64 (0.22, 1.89)	0.57 (0.13,2.55)
≥180	0.94 (0.32, 2.80)	1.04 (0.35, 3.08)	1.15 (0.32, 4.14)
Pre-ICH lipid-lowering drugs use	0.74 (0.41, 1.34)	0.70 (0.38, 1.29)	0.84 (0.43,1.66)
**3-month mRS** **>2**			
<100	1.04 (0.90, 1.20)	0.97 (0.84, 1.13)	0.96 (0.81, 1.13)
<70	1.02 (0.81, 1.28)	0.97 (0.77, 1.22)	0.98 (0.75, 1.29)
70–99	1.30 (1.00, 1.68)	1.26 (0.97, 1.64)	1.27 (0.94, 1.73)
100–109	1.26 (0.90, 1.77)	1.26 (0.89, 1.77)	1.37 (0.92, 2.04)
110–129	1	1	
130–159	0.93 (0.69, 1.26)	0.95 (0.70, 1.29)	1
160–179	1.12 (0.68, 1.82)	1.24 (0.76, 2.04)	1.01 (0.71, 1.44)
≥180	1.01 (0.57, 1.79)	1.21 (0.68, 2.16)	1.24 (0.70, 2.19)
Pre-ICH lipid-lowering drugs use	0.94 (0.70, 1.27)	0.85 (0.63, 1.16)	1.03 (0.54, 1.97)
**Initial NIHSS score** **>15**			
<100	1.36 (1.06, 1.75)	1.35 (1.05, 1.73)	1.35 (1.13, 1.62)
<70	1.65 (1.26, 2.16)	1.64 (1.25, 2.15)	1.53 (1.12, 2.09)
70–99	1.34 (1.16, 1.56)	1.32 (1.14, 1.54)	1.99 (1.42, 2.79)
100–109	1.15 (0.80, 1.64)	1.15 (0.80, 1.63)	1.20 (0.78, 1.86)
110–129	1	1	1
130–159	1.05 (0.75, 1.47)	1.06 (0.76, 1.48)	1.38 (0.93, 2.06)
160–179	1.24 (0.74, 2.05)	1.26 (0.76, 1.48)	1.61 (0.86, 3.00)
≥180	1.02 (0.55, 1.90)	1.06 (0.57, 1.98)	1.17 (0.56, 2.46)
Pre-ICH lipid-lowering drugs use	0.87 (0.63, 1.20)	0.85 (0.61, 1.16)	0.95 (0.65, 1.38)

As reported in Table 6, the Cox proportional hazards models showed that several independent variables were related to 3 months mortality. These included age (OR, 1.01; 95% CI: 1.00–1.02), warfarin (OR, 3.53; 95% CI: 1.31–9.53), glucose (OR, 1.004; 95% CI: 1.001–1.006), LDL-C (OR, 0.99; 95% CI: 0.99–0.996), Creatinine (OR, 1.14; 95% CI: 1.02–1.28), NIHSS (OR, 1.01; 95% CI: 1.01–1.02), GCS (OR, 0.72; 95% CI: 0.68–0.75), hematoma enlargement (OR, 5.37; 95% CI: 2.73–10.6), and surgery for ICH (OR, 0.52; 95% CI: 0.33–0.83).

**Table 6:** Univariate and multivariate logistic regression analyses evaluating the association of baseline characteristics with the likelihood of 3-month mortality.

**Variable**	**Univariate logistic regression analysis**		**Multivariate logistic regression analysis**	
	**OR (95% CI)**	***P*-value**	**OR (95% CI)**	***P*-value**
Age (years)	1.01 (1.00, 1.01)	<0.001	1.01 (1.00, 1.02)	0.049
Sex (%, female)	1.09 (0.99, 1.21)	0.06	1.02 (0.65, 1.60)	0.93
Hypertension	0.61 (0.55, 0.67)	<0.001	0.63 (0.37, 1.07)	0.09
Diabetes	1.25 (1.13, 1.39)	<0.001	0.81 (0.48, 1.36)	0.43
Heart disease	1.54 (1.37, 1.73)	<0.001	1.50 (0.94, 2.37)	0.09
Previous stroke	1.09 (0.97, 1.23)	0.16	1.14 (0.74, 1.77)	0.55
Uremia	3.89 (3.25, 4.65)	<0.001	1.06 (0.39, 2.86)	0.92
Alcoholism	0.91 (0.81, 1.03)	0.13	1.57 (0.97, 2.55)	0.07
Smoking	0.89 (0.80, 0.98)	0.02	0.83 (0.53, 1.30)	0.41
Aspirin	1.00 (0.83, 1.20)	0.99	0.73 (0.34, 1.60)	0.44
Aggrenox	0.51 (0.16, 1.68)	0.27	0.84 (0.09, 8.34)	0.88
Ticlopidine	1.17 (0.52, 2.64)	0.71	-	-
Clopidogrel	2.04 (1.43, 2.92)	<0.001	0.54 (0.10, 3.12)	0.49
Warfarin	1.89 (1.43, 2.49)	<0.001	3.53 (1.31, 9.53)	0.01
Anti H/T drug	0.95 (0.86, 1.04)	0.26	0.87 (0.56, 1.34)	0.52
Anti DM drug	1.17 (1.03, 1.33)	0.02	0.53 (0.27, 1.05)	0.07
Lipid lowering drug	0.79 (0.60, 1.05)	0.10	0.81 (0.30, 2.19)	0.68
Admission SBP	1.00 (1.003, 1.005)	<0.001	1.01 (1.00, 1.01)	0.15
Admission DBP	1.00 (0.99, 1.00)	0.17	1.00 (0.99, 1.01)	0.81
Admission glucose	1.01 (1.01, 1.01)	<0.001	1.004 (1.001,1.006)	0.001
Admission platelets	1.00 (0.99, 1.00)	<0.001	1.00 (0.99, 1.00)	0.17
Admission LDL-C	0.99 (0.99, 0.996)	<0.001	0.99 (0.99, 0.996)	0.001
Admission HDL	0.99 (0.993, 0.999)	0.008	0.99 (0.99, 1.00)	0.11
Admission hemoglobin A1c	0.84 (0.82, 0.86)	<0.001	0.98 (0.89, 1.08)	0.66
Admission creatinine	1.19 (1.17, 1.22)	<0.001	1.14 (1.02, 1.28)	0.02
NIHSS score	1.02 (1.017, 1.019)	<0.001	1.01 (1.01, 1.02)	<0.001
Initial GCS	0.70 (0.68, 0.72)	<0.001	0.72 (0.68, 0.75)	<0.001
Hematoma enlargement	4.22 (2.91, 6.14)	<0.001	5.37 (2.73, 10.6)	<0.001
Surgery for ICH	2.06 (1.59, 2.66)	<0.001	0.52 (0.33, 0.83)	0.006

## Discussion

Low cholesterol, commonly encountered among Taiwanese, has been overlooked as a health risk or a warning sign. In the present study, we described the association between serum LDL-C levels, and ICH mortality by using of 2 data sets. The main finding of our study was that low levels of LDL-C are independently associated with an increased risk of death in patients with ICH (aHR, 1.56; 95% CI, 1.13–2.16), especially in LDL-C levels lower than 70 mg/dL (aHR, 2.53; 95% CI, 1.50–4.26). Compared to patients with LDL 110–129 mg/dL, those with LDL <100 mg/dL had higher 3-month mortality (aOR, 1.84; 95% CI, 1.28–2.63). They were more likely to have hematoma enlargement, and initial NIHSS >15. The effects of lipid-lowering drugs were negligible since only a small percentage of the ICH population was taking them at per-ICH stage.

Many epidemiological studies including the Framingham Heart Study (14), the Multiple Risk Factor Intervention Trial (15), the Coronary Primary Prevention Study (16), and the Helsinki Heart Study (17), have suggested that high cholesterol levels are associated with myocardial infarction and ischemic stroke. The 2013 guidelines for managing blood cholesterol from the American College of Cardiology and the American Heart Association state that lowering cholesterol levels reduces cardiovascular events. This establishes a central, causal role of atherogenic cholesterol-containing lipoprotein particles, particularly LDL-C, in cardiovascular events (18). The most recent 2018 guidelines advocate for a “lower is better” approach to LDL-C. They suggest that an optimal LDL-C level at or below 100 mg/dL lowers the rate of heart disease and stroke (19). This emphasis on low LDL-C is mainly to prevent heart disease.

As Taiwan have fewer deaths due to cardiovascular disease, the benefits of low LDL are far less than in Western countries (20). In 2000, coronary heart disease accounted for 29.6% of the total death rate in the United States while in Taiwan, it accounted for 8.5% (21, 22). The prevalence of LDL with 160 mg/dL or higher was 33% in the United States and only 12.8% in Taiwan (23, 24). The overall difference in proportion was >3 times, and the difference in death rate was more than two times, showcasing that Taiwanese have a lower coronary heart disease mortality rate compared to Westerners and a low prevalence of high cholesterol. On average, the Taiwan population has lower cholesterol and LDL than the Western population, therefore high LDL is not as much of a concern in Taiwan and statins are not equally effective. Therefore, the guidelines were based on Western population and should be applied with great caution in Taiwan's population.

An accumulation of LDL-C in the tunica intima is an important step in the initiation of atherosclerosis (6). Serum cholesterol levels can help maintain the integrity of vascular vessels. Lower cholesterol levels may modify platelet aggregability and decrease the resistance of the vascular wall (25). These effects can lead vessels to rupture and cause ICH. In addition, low LDL-C is associated with a higher risk of some cancers, especially liver cancer, where cholesterol is synthesized. However, the causative direction of this association is not clear, with some arguing that cancer patients had lower cholesterol and not the other way around. Therefore, the “lower the better” approach toward LDL may not be suitable for Taiwanese.

A link between ICH, but not ischemic stroke, and low cholesterol or LDL is well known (26–38). Higher levels of LDL-C seem to be associated with a lower risk of ICH. A previous study by Sturgeon et al. also showed that lower LDL-C and lower triglycerides were risk factors for ICH (26). In a meta-analysis by Wang et al., total cholesterol levels were inversely associated with the risk of ICH (27). Therefore, low LDL-C has been proposed as a risk factor for ICH.

Other studies have suggested that hypertension are bigger risk factors for ICH, more so than low LDL-C (29–32). However, care must be taken in extrapolating from these results because they lack defined LDL-C levels.

Although these results suggest that lower LDL-C at admission is an independent predictor of higher mortality, this evidence should be interpreted with caution because cohorts are Taiwanese (Han Chinese). Taiwanese has lower cholesterol than the Western population, and do not really reflect all East Asian race/ethnicities. Therefore, further research in this area is necessary to determine the best target range of LDL-C levels, especially in patients with atherosclerotic disease who might be at a higher baseline risk of ICH.

Unlike our current study, previous studies have only reported a trend between low LDL-C concentrations and high ICH incidence or mortality (6, 32–36). For example, higher levels of LDL-C at admission were independently (*p* < 0.001) associated with a lower likelihood of in-hospital mortality (OR per 10 mg/dL increase 0.68, 95% CI: 0.57–0.80) in multivariable logistic regression models (6). In another cohort study, LDL-C levels <95mg/dL emerged as an independent predictor of 3-month mortality (OR, 6.34; 95% CI, 1.29 to −31.3; *P* = 0.023) (29). In our study, we showed that LDL-C levels were independently associated with an increased risk of mortality in patients with ICH, especially when LDL-C was lower than 70 mg/dL. A large study from Korea and extensive analyses from Japan essentially support our conclusion (39, 40). Researchers from one study determined the harms of low cholesterol so robust that “their data cast doubt on the scientific justification for lowering cholesterol to very low levels in elderly people” (41). Our results, and other similar findings from previous studies, would suggest the “lower is better” view of LDL-C is not always correct. LDL-C and triglyceride levels drop at the onset of acute illness and return to normal during recovery. More importantly, hypocholesterolemia is not only a marker for the disease severity, but it may also predispose critically ill patients to sepsis and adrenal failure, and may carry a significantly increased risk of mortality (42). While the causal role of cholesterol in mortality may vary, our findings reveal that a high prevalence of low cholesterol is not a cause for celebration, but it could actually be a strong risk factor for mortality. This study suggests that the efforts to keep cholesterol as low as possible may not improve life expectancy but may cause harm.

However, the present study has several limitations. First, it was based on a single LDL-C measurement upon admission. LDL and HDL showed a significant diurnal variation: lowest values were seen early in the morning followed by an increase before breakfast, with the highest levels during the afternoon in most cases. Fluctuations in LDL-C following admission could have a significant impact on the mortality rate, but information on this important aspect was not available within the MJ or TSR dataset. Furthermore, we included all patients that presented at the hospital within 10 days of symptom onset. Thus, the latency period from ICH onset to LDL-C sampling could vary from minutes to 10 days. Secondly, ICH may cause a severe disability but not death most of the time. In this case, the higher risk of death may contribute from other factors, i.e., baseline health condition, complication of ICH and the socioeconomic status. Therefore, ICH mortality maybe underestimation. Thirdly, factors independently associated with mortality were the Glasgow Coma Scale score, age > 80 years, infratentorial origin of ICH, and ICH volume. If hematoma expansion happened in patients with ICH, we noticed a higher neurologic impairment and mortality rate. Our result hint low LDL was associated with the development of hematoma expansion in the setting of spontaneous ICH. We have controlled for several important confounders, but unknown confounders or misclassification of confounders by self-reporting instruments may have resulted in residual confounding. These include the volume and the location of blood clots. Thus, it was not taken in account in the analysis. Fourthly, low LDL-C due to genetics or a marker of an underlying nutritional is unclear. Acute high-fat feeding increased the level of total and LDL cholesterol, significantly reduced HDL cholesterol, and worsened the outcome following ischemic stroke. Healthy low-carbohydrate-diet and low-fat-diet were associated with lower total mortality (43, 44). Those were not adjusted in the analysis. Finally, MJ cohort is a private fee-for-service company offering comprehensive health screening programs. All participants are membership based and may have slightly higher socioeconomic status than the general Taiwan population.

## Conclusion

In conclusion, our study suggests that lower serum LDL-C level independently predicts higher mortality after ICH in Taiwanese Cohorts, especially when LDL-C levels were <100 mg/dL. Although these results suggest that lower LDL-C at admission is an independent predictor of higher mortality, this evidence should be interpreted with caution because cohorts are Taiwanese (Han Chinese). Taiwanese has lower cholesterol than the Western population, and do not really reflect all East Asian race/ethnicities. Therefore, further research in this area is necessary to determine the best target range of LDL-C levels, especially in patients with atherosclerotic disease who might be at a higher baseline risk of ICH.

## Data Availability Statement

The data analyzed in this study were obtained from two independent data sets, the Mei-Jhao (MJ) Health Survey Data and Taiwan Stroke Registry (TSR). The MJ Cohort is available to the worldwide research community and offers collaboration (Authorization Code: MJHRFB2014001C). Applicants for data access should contact the MJ Health Research Foundation (http://www.mjhrf.org/main/page/release2/en/#release01). The TSR data that support the findings of this study are available from Taiwan Stroke Registry, but restrictions apply to the availability of these data, which were used under license for the current study, and so are not publicly available. Data are however available from the authors upon reasonable request and with permission of Taiwan Stroke Registry (taiwanstrokeregistry@gmail.com).

## Ethics Statement

The studies involving human participants were reviewed and approved by China Medical University and the Institutional Review Boards (IRB) of the collaborating hospitals (CMUH104-REC2-115). The patients/participants provided their written informed consent to participate in this study.

## List of TSR Investigators

**Chung-Hsiang Liu**, Division of Neurology, China Medical University Hospital, Taichung, Taiwan; **Wei-Shih Huang**, Division of Neurology, China Medical University Hospital, Taichung, Taiwan; **Chung-Ta Lu**, Division of Neurology, China Medical University Hospital, Taichung, Taiwan; **Tzung-Chang Tsai**, Division of Neurology, China Medical University Hospital, Taichung, Taiwan; **Chun-Hung Tseng**, Division of Neurology, China Medical University Hospital, Taichung, Taiwan; **Kang-Hsu Lin**, Division of Neurology, China Medical University Hospital, Taichung, Taiwan; **Woei-Cherng Shyn**, Division of Neurology, China Medical University Hospital, Taichung, Taiwan; **Yu-Wan Yang**, Division of Neurology, China Medical University Hospital, Taichung, Taiwan; **Yen-Liang Liu**, Division of Neurology, China Medical University Hospital, Taichung, Taiwan; **Der-Yang Cho**, Division of Neurology, China Medical University Hospital, Taichung, Taiwan; **Chun-Chung Chen**, Division of Neurology, China Medical University Hospital, Taichung, Taiwan; **Shih-Pin Hsu**, School of Medicine, I-Shou University, Kaohsiung, Taiwan; **Han-Jung Chen**, School of Medicine, I-Shou University, Kaohsiung, Taiwan; **Cheng-Sen Chang**, School of Medicine, I-Shou University, Kaohsiung, Taiwan; **Hung-Chang Kuo**, School of Medicine, I-Shou University, Kaohsiung, Taiwan; **Lian-Hui Lee**, School of Medicine, I-Shou University, Kaohsiung, Taiwan; **Huan-Wen Tsui**, School of Medicine, I-Shou University, Kaohsiung, Taiwan; **Jung-Chi Tsou**, School of Medicine, I-Shou University, Kaohsiung, Taiwan; **Yan-Tang Wang**, School of Medicine, I-Shou University, Kaohsiung, Taiwan; **Yi-Cheng Tai**, School of Medicine, I-Shou University, Kaohsiung, Taiwan; **Kun-Chang Tsai**, School of Medicine, I-Shou University, Kaohsiung, Taiwan; **Yen-Wen Chen**, School of Medicine, I-Shou University, Kaohsiung, Taiwan; **Kang Lu**, School of Medicine, I-Shou University, Kaohsiung, Taiwan; **Po-Chao Liliang**, School of Medicine, I-Shou University, Kaohsiung, Taiwan; **Yu-Tun Tsai**, School of Medicine, I-Shou University, Kaohsiung, Taiwan; **Cheng-Loong Liang**, School of Medicine, I-Shou University, Kaohsiung, Taiwan; **Kuo-Wei Wang**, School of Medicine, I-Shou University, Kaohsiung, Taiwan; **Jui-Sheng Chen**, School of Medicine, I-Shou University, Kaohsiung, Taiwan; **Po-Yuan Chen**, School of Medicine, I-Shou University, Kaohsiung, Taiwan; **Cien-Leong Chye**, School of Medicine, I-Shou University, Kaohsiung, Taiwan; **Wei-Jie Tzeng**, School of Medicine, I-Shou University, Kaohsiung, Taiwan; **Pei-Hua Wu**, School of Medicine, I-Shou University, Kaohsiung, Taiwan; **Chih-Hung Chen**, Department of Neurology, National Cheng Kung University Hospital, College of Medicine, National Cheng Kung University, Tainan, Taiwan; **Han-Chieh Hsieh**, Department of Neurology, National Cheng Kung University Hospital, College of Medicine, National Cheng Kung University, Tainan, Taiwan; **Hui-Chen Su**, Department of Neurology, National Cheng Kung University Hospital, College of Medicine, National Cheng Kung University, Tainan, Taiwan; **Yu-Shan Lee**, Neurological Institute, Taichung Veterans General Hospital, Taichung, Taiwan; **Hsin-Yi Chi**, Department of Neurology, Show Chwan Memorial Hospital, Changhua, Taiwan; **Chou-Hsiung Pan**, Department of Neurology, Show Chwan Memorial Hospital, Changhua, Taiwan; **Po-Chi Chan**, Department of Neurology, Show Chwan Memorial Hospital, Changhua, Taiwan; **Min-Hsien Hsu**, Department of Neurology, Show Chwan Memorial Hospital, Changhua, Taiwan; **Ya-Ying Wu**, Department of Neurology, Show Chwan Memorial Hospital, Changhua, Taiwan; **Zhi-Zang Huang**, Department of Neurology, Show Chwan Memorial Hospital, Changhua, Taiwan; **Hai-Ming Shoung**, Department of Neurology, Show Chwan Memorial Hospital, Changhua, Taiwan; **Yi-Chen Lo**, Department of Neurology, Show Chwan Memorial Hospital, Changhua, Taiwan; **Fu-Hwa Wang**, Department of Neurology, Show Chwan Memorial Hospital, Changhua, Taiwan; **Chien-Chung Chen** (Principal Investigator), Department of Neurology, St. Martin De Porres Hospital, Chiayi, Taiwan; **Yu-Tai Tsai**, Department of Neurology, St. Martin De Porres Hospital, Chiayi, Taiwan; **Ko-Yi Wang**, Department of Neurology, St. Martin De Porres Hospital, Chiayi, Taiwan; **Tzu-Hsuan Huang**, Department of Neurology, Chang Bing Show Chwan Memorial Hospital, Changhua, Taiwan; **Chao-Nan Yang**, Department of Neurology, Chang Bing Show Chwan Memorial Hospital, Changhua, Taiwan; **Chao-Hsien Hung**, Department of Neurology, Chang Bing Show Chwan Memorial Hospital, Changhua, Taiwan; **Ian Shih**, Department of Neurology, Chang Bing Show Chwan Memorial Hospital, Changhua, Taiwan; **Hsin-Yi Kao**, Department of Neurology, Tainan Municipal An-Nan Hospital-China Medical University, Tainan, Taiwan; **Chien-Jung Lu**, Department of Neurology, En Chu Kong Hospital, Xinbei, Taiwan; **Cheng-Huai Lin**, Department of Neurology, En Chu Kong Hospital, Xinbei, Taiwan; **Chieh-Cheng Huang**, Department of Neurology, En Chu Kong Hospital, Xinbei, Taiwan; **Chang-Hsiu Liu**, Department of Neurology, En Chu Kong Hospital, Xinbei, Taiwan; **Hoi-Fong Chan**, Department of Neurology, En Chu Kong Hospital, Xinbei, Taiwan; **Ping-Kun Chen**, Department of Neurology, Lin Shin Hospital, Taichung, Taiwan; **Pai-Yi Chiu**, Department of Neurology, Lin Shin Hospital, Taichung, Taiwan; **Jiann-Chyun Lin**, Department of Neurology, Tri-Service General Hospital, National Defense Medical Center, Taipei, Taiwan; **Yaw-Don Hsu**, Department of Neurology, Tri-Service General Hospital, National Defense Medical Center, Taipei, Taiwan; **Jong-Chyou Denq**, Department of Neurology, Tri-Service General Hospital, National Defense Medical Center, Taipei, Taiwan; **Giia-Sheun Peng**, Department of Neurology, Tri-Service General Hospital, National Defense Medical Center, Taipei, Taiwan; **Chang-Hung Hsu**, Department of Neurology, Tri-Service General Hospital, National Defense Medical Center, Taipei, Taiwan; **Chun-Chieh Lin**, Department of Neurology, Tri-Service General Hospital, National Defense Medical Center, Taipei, Taiwan; **Che-Hung Yen**, Department of Neurology, Tri-Service General Hospital, National Defense Medical Center, Taipei, Taiwan; **Chun-An Cheng**, Department of Neurology, Tri-Service General Hospital, National Defense Medical Center, Taipei, Taiwan; **Yueh-Feng Sung**, Department of Neurology, Tri-Service General Hospital, National Defense Medical Center, Taipei, Taiwan; **Yuan-Liang Chen**, Department of Neurology, Tri-Service General Hospital, National Defense Medical Center, Taipei, Taiwan; **Ming-Tung Lien**, Department of Neurology, Tri-Service General Hospital, National Defense Medical Center, Taipei, Taiwan; **Chung-Hsing Chou**, Department of Neurology, Tri-Service General Hospital, National Defense Medical Center, Taipei, Taiwan; **Chia-Chen Liu**, Department of Neurology, Tri-Service General Hospital, National Defense Medical Center, Taipei, Taiwan; **Fu-Chi Yang**, Department of Neurology, Tri-Service General Hospital, National Defense Medical Center, Taipei, Taiwan; **Yi-Chung Wu**, Department of Neurology, Tri-Service General Hospital, National Defense Medical Center, Taipei, Taiwan; **An-Chen Tso**, Department of Neurology, Tri-Service General Hospital, National Defense Medical Center, Taipei, Taiwan; **Yu- Hua Lai**, Department of Neurology, Tri-Service General Hospital, National Defense Medical Center, Taipei, Taiwan; **Chun-I Chiang**, Department of Neurology, Tri-Service General Hospital, National Defense Medical Center, Taipei, Taiwan; **Chia-Kuang Tsai**, Department of Neurology, Tri-Service General Hospital, National Defense Medical Center, Taipei, Taiwan; **Meng-Ta Liu**, Department of Neurology, Tri-Service General Hospital, National Defense Medical Center, Taipei, Taiwan; **Ying-Che Lin**, Department of Neurology, Tri-Service General Hospital, National Defense Medical Center, Taipei, Taiwan; **Yu-Chuan Hsu**, Department of Neurology, Tri-Service General Hospital, National Defense Medical Center, Taipei, Taiwan; **Jiu-Haw Yin**, Department of Neurology, Cheng Hsin General Hospital, Taipei, Taiwan; **Chung-JenWang**, Department of Neurology, Cheng Hsin General Hospital, Taipei, Taiwan; **Kai-ChenWang**, Department of Neurology, Cheng Hsin General Hospital, Taipei, Taiwan; **Li-Mei Chen**, Department of Neurology, Cheng Hsin General Hospital, Taipei, Taiwan; **Jong-Chyou Denq**, Department of Neurology, Cheng Hsin General Hospital, Taipei, Taiwan; **Hou-Chang Chiu**, Department of Neurology, Shin Kong Wu Ho Su Memorial Hospital, Taipei, Taiwan; **Wei-Hung Chen**, Department of Neurology, Shin Kong Wu Ho Su Memorial Hospital, Taipei, Taiwan; **Chyi-Huey Bai**, Department of Neurology, Shin Kong Wu Ho Su Memorial Hospital, Taipei, Taiwan; **Tzu-Hsuan Huang**, Department of Neurology, Shin Kong Wu Ho Su Memorial Hospital, Taipei, Taiwan; **Chi-Ieong Lau**, Department of Neurology, Shin Kong Wu Ho Su Memorial Hospital, Taipei, Taiwan; **Ya-Ying Wu**, Department of Neurology, Shin Kong Wu Ho Su Memorial Hospital, Taipei, Taiwan; **Hsu-Ling Yeh**, Department of Neurology, Shin Kong Wu Ho Su Memorial Hospital, Taipei, Taiwan; **Anna Chang**, Department of Neurology, Shin Kong Wu Ho Su Memorial Hospital, Taipei, Taiwan; Department of Neurology, Cheng Hsin General Hospital, Taipei, Taiwan; **Jiann-Shing Jeng**, Department of Neurology, National Taiwan University Hospital, Taiwan; **Sung-Chun Tang**, Department of Neurology, National Taiwan University Hospital, Taiwan; **Li-Kai Tsai**, Department of Neurology, National Taiwan University Hospital, Taiwan; **Shin-Joe Yeh**, Department of Neurology, National Taiwan University Hospital, Taiwan; **Ching-Huang Lin**, Department of Neurology, Kaohsiung Veterans General Hospital, Taiwan; **Cheng-Chang Yen**, Department of Neurology, Kaohsiung Veterans General Hospital, Taiwan; **Ruey-Tay Lin**, Department of Neurology, Kaohsiung Medical University Chung-Ho Memorial Hospital, Taiwan; **Chun-Hung Chen**, Department of Neurology, Kaohsiung Medical University Chung-Ho Memorial Hospital, Taiwan; **Gim-Thean Khor**, Department of Neurology, Kaohsiung Medical University Chung-Ho Memorial Hospital, Taiwan; **A-Ching Chao**, Department of Neurology, Kaohsiung Medical University Chung-Ho Memorial Hospital, Taiwan; **Hsiu-Fen Lin**, Department of Neurology, Kaohsiung Medical University Chung-Ho Memorial Hospital, Taiwan; **Poyin Huang**, Department of Neurology, Kaohsiung Medical University Chung-Ho Memorial Hospital, Taiwan; **Huey-Juan Lin**, Department of Neurology, Chi Mei Medical Center, Taiwan; **Der-Shin Ke**, Department of Neurology, Chi Mei Medical Center, Taiwan; **Chia-Yu Chang**, Department of Neurology, Chi Mei Medical Center, Taiwan; **Poh-Shiow Yeh**, Department of Neurology, Chi Mei Medical Center, Taiwan; **Kao-Chang Lin**, Department of Neurology, Chi Mei Medical Center, Taiwan; **Tain-Junn Cheng**, Department of Neurology, Chi Mei Medical Center, Taiwan; **Chih-Ho Chou**, Department of Neurology, Chi Mei Medical Center, Taiwan; **Chun-Ming Yang**, Department of Neurology, Chi Mei Medical Center, Taiwan; **Hsiu-Chu Shen**, Department of Neurology, Chi Mei Medical Center, Taiwan; **An-Chih Chen**, Department of Neurology, Chung Shan Medical University Hospital, Taiwan; **Shih-Jei Tsai**, Department of Neurology, Chung Shan Medical University Hospital, Taiwan; **Tsong-Ming Lu**, Department of Neurology, Chung Shan Medical University Hospital, Taiwan; **Sheng-Ling Kung**, Department of Neurology, Chung Shan Medical University Hospital, Taiwan; **Mei-Ju Lee**, Department of Neurology, Chung Shan Medical University Hospital, Taiwan; **Hsi-Hsien Chou**, Department of Neurology, Chung Shan Medical University Hospital, Taiwan; **Siu-Pak Lee**, Department of Neurology, Far Eastern Memorial Hospital, Taiwan; **Ming-Hui Sun**, Department of Neurology, Kuang Tien General Hospital, Taiwan; **Li-Ying Ke**, Department of Neurology, Kuang Tien General Hospital, Taiwan; **Sheng-Feng Sung** (Principal Investigator), Department of Neurology, Ditmanson Medical Foundation Chia-Yi Christian Hospital, Taiwan; **Cheung-Ter Ong**, Department of Neurology, Ditmanson Medical Foundation Chia-Yi Christian Hospital, Taiwan; **Chi-Shun Wu**, Yung-Chu Hsu, Department of Neurology, Ditmanson Medical Foundation Chia-Yi Christian Hospital, Taiwan; **Yu-Hsiang Su**, Department of Neurology, Ditmanson Medical Foundation Chia-Yi Christian Hospital, Taiwan; **Ling-Chien Hung**, Department of Neurology, Ditmanson Medical Foundation Chia-Yi Christian Hospital, Taiwan; **Tsuey-Ru Chiang**, Department of Neurology, Cathay General Hospital, Taiwan; **Mei-Ching Lee**, Department of Neurology, Cathay General Hospital, Taiwan; **Pai-Hao Huang**, Department of Neurology, Cathay General Hospital, Taiwan; **Sian-King Lie**, Department of Neurology, Cathay General Hospital, Taiwan; **Pin-Wen Liao**, Department of Neurology, Cathay General Hospital, Taiwan; **Jen-Tse Chen**, Department of Neurology, Cathay General Hospital, Taiwan; **Mu-Chien Sun**, Department of Neurology, Changhua Christian Hospital, Taiwan; **Tien-Pao Lai**, Department of Neurology, Changhua Christian Hospital, Taiwan; **Wei-Liang Chen**, Department of Neurology, Changhua Christian Hospital, Taiwan; **Yen-Chun Chen**, Department of Neurology, Changhua Christian Hospital, Taiwan; **Ta-Cheng Chen**, Department of Neurology, Changhua Christian Hospital, Taiwan; **Wen-Fu Wang**, Department of Neurology, Changhua Christian Hospital, Taiwan; **Kwo-Whei Lee**, Department of Neurology, Changhua Christian Hospital, Taiwan; **Chen-Shu Chang**, Department of Neurology, Changhua Christian Hospital, Taiwan; **Chien-Hsu Lai**, Department of Neurology, Changhua Christian Hospital, Taiwan; **Siao-Ya Shih**, Department of Neurology, Changhua Christian Hospital, Taiwan; **Chieh-Sen Chuang**, Department of Neurology, Changhua Christian Hospital, Taiwan; **Yen-Yu Chen**, Department of Neurology, Changhua Christian Hospital, Taiwan; **Chien-Min Chen**, Department of Neurology, Changhua Christian Hospital, Taiwan; **Shinn-Kuang Lin**, Department of Neurology, Taipei Tzuchi Hospital, Buddhist Tzuchi Medical Foundation, Taiwan; **Yu-Chin Su**, Department of Neurology, Taipei Tzuchi Hospital, Buddhist Tzuchi Medical Foundation, Taiwan; **Cheng-Lun Hsiao**, Department of Neurology, Taipei Tzuchi Hospital, Buddhist Tzuchi Medical Foundation, Taiwan; **Fu-Yi Yang**, Department of Neurology, Taipei Tzuchi Hospital, Buddhist Tzuchi Medical Foundation, Taiwan; **Chih-Yang Liu**, Department of Neurology, Taipei Tzuchi Hospital, Buddhist Tzuchi Medical Foundation, Taiwan; **Han-Lin Chiang**, Department of Neurology, Taipei Tzuchi Hospital, Buddhist Tzuchi Medical Foundation, Taiwan; **Ser-Chen Fu**, Department of Neurology, Taipei Tzuchi Hospital, Buddhist Tzuchi Medical Foundation, Taiwan; **Chun-Yuan Chang**, Department of Neurology, Min Sheng General Hospital, Taiwan; **I-sheng Lin**, Department of Neurology, Min Sheng General Hospital, Taiwan; **Chung-Hsien Chien**, Department of Neurology, Min Sheng General Hospital, Taiwan; **Yang-Chuang Chang**, Department of Neurology, Min Sheng General Hospital, Taiwan; **Yu-Jen Hsiao**, Department of Neurology, National Taiwan University Hospital Yunlin Branch, Taiwan; **Chen-Wen Fang**, Department of Neurology, National Taiwan University Hospital Yunlin Branch, Taiwan; **Yu-Wei Chen**, Department of Neurology, Landseed Hospital, Taiwan; **Kuo-Ying Lee**, Department of Neurology, Landseed Hospital, Taiwan; **Yun-Yu Lin**, Department of Neurology, Landseed Hospital, Taiwan; **Chen-Hua Li**, Department of Neurology, Landseed Hospital, Taiwan; **Hui-Fen Tsai**, Department of Neurology, Landseed Hospital, Taiwan; **Chuan-Fa Hsieh**, Department of Neurology, Landseed Hospital, Taiwan; **Chih-Dong Yang**, Department of Neurology, Landseed Hospital, Taiwan; **Shiumn-Jen Liaw**, Department of Neurology, Landseed Hospital, Taiwan; **How-Chin Liao**, Department of Neurology, Landseed Hospital, Taiwan; **Shoou-Jeng Yeh** (Principal Investigator), Department of Neurology, Cheng Ching General Hospital, Taiwan; Ling-Li Wu, Department of Neurology, Cheng Ching General Hospital, Taiwan; **Liang-Po Hsieh**, Department of Neurology, Cheng Ching General Hospital, Taiwan; **Yong-Hui Lee**, Department of Neurology, Cheng Ching General Hospital, Taiwan; **Chung-Wen Chen**, Department of Neurology, Cheng Ching General Hospital, Taiwan; **Chih-Shan Hsu**, Department of Neurology, China Medical University Beigang Hospital, Taiwan; **Ye-Jian-Jhih**, Department of Neurology, China Medical University Beigang Hospital, Taiwan; **Hao-Yu Zhuang**, Department of Neurology, China Medical University Beigang Hospital, Taiwan; **Yan-Hong Pan**, Department of Neurology, China Medical University Beigang Hospital, Taiwan; **Shin-An Shih**, Department of Neurology, China Medical University Beigang Hospital, Taiwan; **Chin-I Chen**, Department of Neurology, Taipei Medical University -Wan Fang Hospital, Taiwan; **Jia-Ying Sung**, Department of Neurology, Taipei Medical University -Wan Fang Hospital, Taiwan; **Hsing-Yu Weng**, Department of Neurology, Taipei Medical University -Wan Fang Hospital, Taiwan; **Hao-Wen Teng**, Department of Neurology, Taipei Medical University -Wan Fang Hospital, Taiwan; **Jing-Er Lee**, Department of Neurology, Taipei Medical University -Wan Fang Hospital, Taiwan; **Chih-Shan Huang**, Department of Neurology, Taipei Medical University -Wan Fang Hospital, Taiwan; **Shu-Ping Chao**, Department of Neurology, Taipei Medical University -Wan Fang Hospital, Taiwan; **Rey-Yue Yuan**, Department of Neurology, Taipei Medical University Hospital, Taiwan; **Jau- Jiuan Sheu**, Department of Neurology, Taipei Medical University Hospital, Taiwan; **Jia-Ming Yu**, Department of Neurology, Taipei Medical University Hospital, Taiwan; **Chun-Sum Ho**, Department of Neurology, Taipei Medical University Hospital, Taiwan; **Ting-Chun Lin**, Department of Neurology, Taipei Medical University Hospital, Taiwan; **Shih-Chieh Yu**, Department of Neurology, Kuang Tien General Hospital Dajia Division, Taiwan; **Jiunn-Rong Chen**, Department of Neurology, Changhua Christian Hospital Yunlin Branch, Taiwan; **Song-Yen Tsai**, Department of Neurology, Changhua Christian Hospital Yunlin Branch, Taiwan; **Hung-Pin Tseng**, Department of Neurology, Lotung Poh Ai Hospital, Taiwan; **Chin-Hsiung Liu**, Department of Neurology, Lotung Poh Ai Hospital, Taiwan; **Chun-Liang Lin**, Department of Neurology, Lotung Poh Ai Hospital, Taiwan; **Hung-Chih Lin**, Department of Neurology, Lotung Poh Ai Hospital, Taiwan; **Pi-Tzu Chen**, Department of Neurology, Lotung Poh Ai Hospital, Taiwan; **Chaur-Jong Hu**, Department of Neurology, Taipei Medical University - Shuang Ho Hospital, Taiwan; **Nai-Fang Chi**, Department of Neurology, Taipei Medical University - Shuang Ho Hospital, Taiwan; **Lung Chan**, Department of Neurology, Taipei Medical University - Shuang Ho Hospital, Taiwan; **Chang-Ming Chern**, Department of Neurology, Taipei Veterans General Hospital & National Yang-Ming University School of Medicine, Taiwan; **Chun-Jen Lin**, Department of Neurology, Taipei Veterans General Hospital & National Yang-Ming University School of Medicine, Taiwan; **Shuu-Jiun Wang**, Department of Neurology, Taipei Veterans General Hospital & National Yang-Ming University School of Medicine, Taiwan; **Li-Chi Hsu**, Department of Neurology, Taipei Veterans General Hospital & National Yang-Ming University School of Medicine, Taiwan; **Wen-Jang Wong**, Department of Neurology, Taipei Veterans General Hospital & National Yang-Ming University School of Medicine, Taiwan; **I-Hui Lee**, Department of Neurology, Taipei Veterans General Hospital & National Yang-Ming University School of Medicine, Taiwan; **Der-Jen Yen**, Department of Neurology, Taipei Veterans General Hospital & National Yang-Ming University School of Medicine, Taiwan; **Ching-Piao Tsai**, Department of Neurology, Taipei Veterans General Hospital & National Yang-Ming University School of Medicine, Taiwan; **Shang-Yeong Kwan**, Department of Neurology, Taipei Veterans General Hospital & National Yang-Ming University School of Medicine, Taiwan; **Bing-Wen Soong**, Department of Neurology, Taipei Veterans General Hospital & National Yang-Ming University School of Medicine, Taiwan; **Shih-Pin Chen**, Department of Neurology, Taipei Veterans General Hospital & National Yang-Ming University School of Medicine, Taiwan; **Kwong-Kum Liao**, Department of Neurology, Taipei Veterans General Hospital & National Yang-Ming University School of Medicine, Taiwan; **Kung-Ping Lin**, Department of Neurology, Taipei Veterans General Hospital & National Yang-Ming University School of Medicine, Taiwan; **Chien Chen**, Department of Neurology, Taipei Veterans General Hospital & National Yang-Ming University School of Medicine, Taiwan; **Din-E Shan**, Department of Neurology, Taipei Veterans General Hospital & National Yang-Ming University School of Medicine, Taiwan; **Jong-Ling Fuh**, Department of Neurology, Taipei Veterans General Hospital & National Yang-Ming University School of Medicine, Taiwan; **Pei-Ning Wang**, Department of Neurology, Taipei Veterans General Hospital & National Yang-Ming University School of Medicine, Taiwan; **Yi-Chung Lee**, Department of Neurology, Taipei Veterans General Hospital & National Yang-Ming University School of Medicine, Taiwan; **Yu-Hsiang Yu**, Department of Neurology, Taipei Veterans General Hospital & National Yang-Ming University School of Medicine, Taiwan; **Hui-Chi Huang**, Department of Neurology, Taipei Veterans General Hospital & National Yang-Ming University School of Medicine, Taiwan; **Jui-Yao Tsai**, Department of Neurology, Taipei Veterans General Hospital & National Yang-Ming University School of Medicine, Taiwan; **Ming-Hsiu Wu**, Department of Neurology, Chi Mei Medical Center, Liouying, Taiwan; **Shi-Cheng Chen**, Szu-Yi Chiang, Department of Neurology, Chi Mei Medical Center, Liouying, Taiwan; **Chiung-Yao Wang**, Department of Neurology, Chi Mei Medical Center, Liouying, Taiwan; **Ming-Chin Hsu**, Department of Neurology, Buddhist Dalin Tzu Chi General Hospital, Taiwan; **Tsang-Shan Chen**, Department of Neurology, Sin-Lau Hospital, Tainan, the Presbyterian Church, Taiwan; **Ping-Keung Yip**, Department of Neurology, Cardinal Tien Hospital, Taiwan; **Vinchi Wang**, Department of Neurology, Cardinal Tien Hospital, Taiwan; **Kaw-ChenWang**, Department of Neurology, Cardinal Tien Hospital, Taiwan; **Chung-Fen Tsai**, Department of Neurology, Cardinal Tien Hospital, Taiwan; **Chao-Ching Chen**, Department of Neurology, Cardinal Tien Hospital, Taiwan; **Chih-Hao Chen**, Department of Neurology, Cardinal Tien Hospital, Taiwan; **Yi-Chien Liu**, Department of Neurology, Cardinal Tien Hospital, Taiwan; **Shao-Yuan Chen**, Department of Neurology, Cardinal Tien Hospital, Taiwan; **Zi-Hao Zhao**, Department of Neurology, Cardinal Tien Hospital, Taiwan; **Zhi-Peng Wei**, Department of Neurology, Cardinal Tien Hospital, Taiwan; **Shey-Lin Wu**, Department of Neurology, Yumin Medical Corporation Yumin Hospital, Taiwan; **Ching-Kuan Liu**, Department of Neurology, Kaohsiung Municipal Hsiao-kang Hospital, Taiwan; **Ryh-Huei Lin**, Department of Neurology, Wei Gong Memorial Hospital, Taiwan; **Ching-Hua Chu**, Department of Neurology, Wei Gong Memorial Hospital, Taiwan; **Sui-Hing Yan**, Department of Neurology, Taipei City Hospital Ren Ai Branch, Taiwan; **Yi-Chun Lin**, Department of Neurology, Taipei City Hospital Ren Ai Branch, Taiwan; **Pei-Yun Chen**, Department of Neurology, Taipei City Hospital Ren Ai Branch, Taiwan; **Sheng-Huang Hsiao**, Department of Neurology, Taipei City Hospital Ren Ai Branch, Taiwan; **Bak-Sau Yip**, Department of Neurology, National Taiwan University Hospital Hsin-Chu Branch, Taiwan; **Pei-Chun Tsai**, Department of Neurology, National Taiwan University Hospital Hsin-Chu Branch, Taiwan; **Ping-Chen Chou**, Department of Neurology, National Taiwan University Hospital Hsin-Chu Branch, Taiwan; **Tsam-Ming Kuo**, Department of Neurology, National Taiwan University Hospital Hsin-Chu Branch, Taiwan; **Yi-Chen Lee**, Department of Neurology, National Taiwan University Hospital Hsin-Chu Branch, Taiwan; **Yi-Pin Chiu**, Department of Neurology, National Taiwan University Hospital Hsin-Chu Branch, Taiwan; **Kun-Chang Tsai**, Department of Neurology, National Taiwan University Hospital Hsin-Chu Branch, Taiwan; **Yi-Sheng Liao**, Department of Neurology, Taichung Hospital, Taiwan.

## Author Contributions

C-PW, Y-CL, H-KW, and CH: designed and conceptualized study, analyzed the data, data acquisition, and drafted the manuscript for intellectual content. Y-TS and C-YH: designed and conceptualized study and analyzed the data. C-HT, P-LC, W-LC, P-YY, C-YW, and M-JT: collection and analysis of data and editing of the manuscript. YS, C-HL, J-TL, T-CL, L-ML, M-CL, C-LL, and J-HL: conception and design of the study and drafting of the manuscript and preparation of figures. All authors contributed to the article and approved the submitted version.

## Funding

This study was supported in part by Taiwan Ministry of Health and Welfare Clinical Trial Center (MOHW109-TDU-B-212-114004), Ministry of Science and Technology (MOST 110-2321-B-039-003), MOST Clinical Trial Consortium for Stroke (MOST 108-2321-B-039-003), and Tseng-Lien Lin Foundation, Taichung, Taiwan.

## Conflict of Interest

The authors declare that the research was conducted in the absence of any commercial or financial relationships that could be construed as a potential conflict of interest.

## Publisher's Note

All claims expressed in this article are solely those of the authors and do not necessarily represent those of their affiliated organizations, or those of the publisher, the editors and the reviewers. Any product that may be evaluated in this article, or claim that may be made by its manufacturer, is not guaranteed or endorsed by the publisher.
