# Supplementary material for: Low-Density Lipoprotein Cholesterol and Mortality in Patients With Intracerebral Hemorrhage in Taiwan
Source: Front Neurol. 2022 Jan 17;12:793471. doi: 10.3389/fneur.2021.793471 (PMC8802633; doi:10.3389/fneur.2021.793471)
Supplement: Supplementary file 1 [file Table_1.docx]

**ORIGINAL RESEARCH**

**Low-density lipoprotein Cholesterol and Mortality in Patients With Intracerebral Hemorrhage in Taiwan**

Chi-Pang Wen^1,2,3^, Yi-Che Lee^4, 5^, Yuan-Ting Sun^6^, Chih-Yuan Huang^7^, Chon-Haw Tsai^8^, Po-Lin Chen^9^, Wei-Lun Chang^10^, Po-Yen Yeh^11^, Cheng-Yu Wei^12^, Ming-Jun Tsai^13^, Yu Sun^14^, Chih-Hao Lin^15^, Jiunn-Tay Lee^16^, Ta-Chang Lai^17^, Li-Ming Lien^18^, Mei-Chen Lin^19, 20^, Cheng-Li Lin^19, 20^, June-Han Lee ^3^, Hao-Kuang Wang^4, 21^, Chung Y. Hsu ^22, 23^ ,Taiwan Stroke Registry Investigators^24^.

^1^ Graduate Institute of Biomedical Sciences, College of Medicine, China Medical University, Taichung, Taiwan.

^2^ Department of Medical Research, China Medical University Hospital, Taichung, Taiwan.

^3^ Institute of Population Health Sciences, National Health Research Institutes, Zhunan, Taiwan

^4^ School of Medicine, I-Shou University, Kaohsiung, Taiwan

^5^ Department of Nephrology, E-Da Hospital, Kaohsiung, Taiwan

^6^ Department of Neurology, National Cheng Kung University Hospital, College of Medicine, National Cheng Kung University, Tainan, Taiwan

^7^ Neurosurgical Service, Department of Surgery, National Cheng Kung University Hospital, Tainan, Taiwan

^8^ Division of Neurology, China Medical University Hospital, Taichung, Taiwan

^9^ Neurological Institute, Taichung Veterans General Hospital, Taichung, Taiwan

^10^ Department of Neurology, Show Chwan Memorial Hospital, Changhua County, Taiwan

^11^ Department of Neurology, St. Martin De Porres Hospital, Chiayi, Taiwan

^12^ Department of Neurology, Chang Bing Show Chwan Memorial Hospital, Changhua County, Taiwan

^13^ Department of Neurology, Tainan Municipal An-Nan Hospital-China Medical University, Tainan, Taiwan

^14^ Neurology, En Chu Kong Hospital, Xinbei City, Taiwan

^15^ Neurology, Lin Shin Hospital, Taichung, Taiwan

^16^ Department of Neurology, Tri-Service General Hospital, Taipei, Taiwan

^17^ Department of Neurology, Cheng Hsin General Hospital, Taipei, Taiwan

^18^ Department of Neurology, Shin Kong Wu Ho Su Memorial Hospital, Taipei, Taiwan

^19^ Management Office for Health Data, China Medical University Hospital, Taichung, Taiwan

^20^ College of Medicine, China Medical University, Taichung, Taiwan

^21^ Department of Neurosurgery, E-Da Hospital, Kaohsiung, Taiwan

^22^ Department of Neurology, China Medical University Hospital, Taichung, Taiwan

^23^ Graduate Institute of Clinical Medical Science, China Medical University, Taichung, Taiwan

^24^ Taiwan Stroke Society, Taiwan

Supply Table 1. Baseline characteristics of the study population in TSR, n (%)

|  | Total | LDL-C |  |  |  |  |  |  |  |  |
| --- | --- | --- | --- | --- | --- | --- | --- | --- | --- | --- |
|  | N=4606 | < 100  N=3396 | < 70  N=2487 | 70-99  N=909 | 100-109  N=281 | 110-129  N=394 | 130-159  N=375 | 160-179  N=96 | ≥ 190  N=64 | P value |
| Sex |  |  |  |  |  |  |  |  |  | 0.39 |
| Women | 1519(33.0) | 1088(32.0) | 803(32.3) | 285(31.4) | 99(35.2) | 137(34.8) | 139(37.1) | 32(33.3) | 24(37.5) |  |
| Men | 3087(67.0) | 2308(68.0) | 1684(67.7) | 624(68.7) | 182(64.8) | 257(65.2) | 236(62.9) | 64(66.7) | 40(62.5) |  |
| Age (years) |  |  |  |  |  |  |  |  |  | <0.001 |
| 20-29 | 32(0.69) | 26(0.77) | 16(0.64) | 10(1.10) | 1(0.36) | 2(0.51) | 2(0.53) | 0(0.00) | 1(1.56) |  |
| 30-39 | 183(3.97) | 114(3.36) | 80(3.22) | 34(3.74) | 13(4.63) | 16(4.06) | 25(6.67) | 9(9.38) | 6(9.38) |  |
| 40-49 | 641(13.9) | 443(13.0) | 326(13.1) | 117(12.9) | 40(14.2) | 63(16.0) | 61(16.3) | 18(18.8) | 16(25.0) |  |
| 50-59 | 1165(25.3) | 850(25.0) | 637(25.6) | 213(23.4) | 81(28.8) | 88(22.3) | 96(25.6) | 31(32.3) | 19(29.7) |  |
| 60-69 | 1022(22.2) | 723(21.3) | 510(20.5) | 213(23.4) | 56(19.9) | 120(30.5) | 90(24.0) | 18(18.8) | 15(23.4) |  |
| >70 | 1563(33.9) | 1240(36.5) | 918(36.9) | 322(35.4) | 90(32.0) | 105(26.7) | 101(26.9) | 20(20.8) | 7(10.9) |  |
| Median (Q1, Q3) | 61.7  (51.9, 72.8) | 62.4(52.5, 73.9) | 62.3(52.6, 74.1) | 62.6(52.3, 73.4) | 59.8(51.3, 72.4) | 61.4(51.6, 69.6) | 59.6(49.6, 70.8) | 55.6(47.1, 64.8) | 53.7(44.7, 62.3) | <0.001 |

Supply Table 2. Comorbidities of the study population in TSR, n (%)

| Medical history | Total | LDL-C |  |  |  |  |  |  |  |  |
| --- | --- | --- | --- | --- | --- | --- | --- | --- | --- | --- |
|  |  | < 100 | < 70 | 70-99 | 100-109 | 110-129 | 130-159 | 160-179 | ≥ 180 | P value |
| History of hypertension |  |  |  |  |  |  |  |  |  | <0.001 |
| No | 783(17.0) | 668(19.7) | 524(21.1) | 144(15.8) | 35(12.5) | 43(10.9) | 25(6.67) | 9(9.38) | 3(4.69) |  |
| Yes | 3823(83.0) | 2728(80.3) | 1963(78.9) | 765(84.2) | 246(87.5) | 351(89.1) | 350(93.3) | 87(90.6) | 61(95.3) |  |
| Admission systolic blood pressure |  |  |  |  |  |  |  |  |  | 0.15 |
| < 120 | 169(3.67) | 146(4.30) | 110(4.42) | 36(3.96) | 5(1.78) | 11(2.79) | 5(1.33) | 1(1.04) | 1(1.56) |  |
| 120-139 | 422(9.16) | 327(9.63) | 234(9.41) | 93(10.2) | 23(8.19) | 33(8.38) | 31(8.27) | 5(5.21) | 3(4.69) |  |
| 140-159 | 793(17.2) | 586(17.3) | 430(17.3) | 156(17.2) | 51(18.2) | 67(17.0) | 63(16.8) | 16(16.7) | 10(15.6) |  |
| ≥ 160 | 3222(70.0) | 2337(68.8) | 1713(68.9) | 624(68.7) | 202(71.9) | 283(71.8) | 276(73.6) | 74(77.1) | 50(78.1) |  |
| History of diabetes |  |  |  |  |  |  |  |  |  | 0.07 |
| No | 3477(75.5) | 2538(74.7) | 1879(75.6) | 659(72.5) | 225(80.1) | 304(77.2) | 283(75.5) | 80(83.3) | 47(73.4) |  |
| Yes | 1129(24.5) | 858(25.3) | 608(24.5) | 250(27.5) | 56(19.9) | 90(22.8) | 92(24.5) | 16(16.7) | 17(26.6) |  |
| HbA1c < 7% | 16(0.35) | 11(0.32) | 9(0.36) | 2(0.22) | 1(0.36) | 2(0.51) | 2(0.53) | 0(0.00) | 0(0.00) | 0.19 |
| 7 ≤ HbA1c < 8 | 20(0.43) | 18(0.53) | 16(0.64) | 2(0.22) | 0(0.00) | 0(0.00) | 0(0.00) | 2(2.08) | 0(0.00) |  |
| HbA1c ≥ 8 | 4570(99.2) | 3367(99.2) | 2462(99.0) | 905(99.6) | 280(99.6) | 392(99.5) | 373(99.5) | 94(97.9) | 64(100.0) |  |
| Heart disease | 693(15.1) | 543(16.0) | 410(16.5) | 133(14.6) | 36(12.8) | 57(14.5) | 44(11.7) | 7(7.29) | 6(9.38) | 0.02 |
| Previous stroke | 793(17.2) | 607(17.9) | 448(18.0) | 159(17.5) | 39(13.9) | 71(18.0) | 53(14.1) | 14(14.6) | 9(14.1) | 0.33 |
| Uremia | 113(2.95) | 92(3.23) | 69(3.22) | 23(3.25) | 5(2.20) | 10(3.22) | 4(1.27) | 1(1.35) | 1(1.85) | 0.50 |
| Alcoholism | 1003(21.8) | 749(22.1) | 499(20.1) | 250(27.5) | 69(24.6) | 87(22.1) | 64(17.1) | 17(17.7) | 17(26.6) | <0.001 |
| Smoking | 1617(37.8) | 1174(37.8) | 801(36.6) | 373(43.4) | 101(37.4) | 162(43.1) | 111(30.5) | 35(38.0) | 34(53.1) | <0.001 |

Supply Table 3. Pre-ICH medications of the study population in TSR, n (%)

| Pre-ICH medications | Total | LDL-C |  |  |  |  |  |  |  |  |
| --- | --- | --- | --- | --- | --- | --- | --- | --- | --- | --- |
|  |  | < 100 | < 70 | 70-99 | 100-109 | 110-129 | 130-159 | 160-179 | ≥ 180 | P value |
| Aspirin | 322(6.99) | 252(7.42) | 188(7.56) | 64(7.04) | 18(6.41) | 24(6.09) | 22(5.87) | 3(3.13) | 3(4.69) | 0.55 |
| Aggrenox | 11(0.24) | 9(0.27) | 8(0.32) | 1(0.11) | 2(0.71) | 0(0.00) | 0(0.00) | 0(0.00) | 0(0.00) | 0.82 |
| Ticlopidine | 16(0.35) | 12(0.35) | 8(0.32) | 4(0.44) | 2(0.71) | 1(0.25) | 1(0.27) | 0(0.00) | 0(0.00) | 0.91 |
| Clopidogrel | 50(1.09) | 38(1.12) | 26(1.05) | 12(1.32) | 3(1.07) | 4(1.02) | 5(1.33) | 0(0.00) | 0(0.00) | 0.87 |
| Warfarin | 75(1.63) | 59(1.74) | 49(1.97) | 10(1.10) | 2(0.71) | 4(1.02) | 6(1.60) | 2(2.08) | 2(3.13) | 0.34 |
| anti H/T drug | 1731(37.6) | 1286(37.9) | 962(38.7) | 324(35.6) | 94(33.5) | 145(36.8) | 148(39.5) | 37(38.5) | 21(32.8) | 0.41 |
| anti DM drug | 637(13.8) | 497(14.6) | 350(14.1) | 147(16.2) | 33(11.7) | 51(12.9) | 44(11.7) | 6(6.25) | 6(9.38) | 0.049 |
| lipid lowering drug | 208(4.52) | 153(4.51) | 105(4.22) | 48(5.28) | 10(3.56) | 15(3.81) | 22(5.87) | 5(5.21) | 3(4.69) | 0.61 |
